# Protection against Doxorubicin-Induced Cardiotoxicity through Modulating iNOS/ARG 2 Balance by Electroacupuncture at PC6

**DOI:** 10.1155/2021/6628957

**Published:** 2021-03-20

**Authors:** Jingya Wang, Lin Yao, Xiaoli Wu, Qi Guo, Shengxuan Sun, Jie Li, Guoqi Shi, Ruth B. Caldwell, R. William Caldwell, Yongjun Chen

**Affiliations:** ^1^South China Research Center for Acupuncture and Moxibustion, Medical College of Acu-Moxi and Rehabilitation, Guangzhou University of Chinese Medicine, Guangzhou, 510006, China; ^2^School of Pharmaceutical Sciences, Guangzhou University of Chinese Medicine, Guangzhou, 510006, China; ^3^Vascular Biology Center, Medical College of Georgia, Augusta University, Augusta, GA, USA; ^4^Department of Pharmacology and Toxicology, Medical College of Georgia, Augusta University, Augusta, GA, USA; ^5^Center for Brain Science and Brain-Inspired Intelligence, Guangdong-Hong Kong-Macao Greater Bay Area, Guangzhou 510515, China

## Abstract

**Background:**

Doxorubicin (DOX) is a commonly used chemotherapeutic drug but is limited in clinical applications by its cardiotoxicity. *Neiguan* acupoint (PC6) is a well-recognized acupoint for the treatment of cardiothoracic disease. However, whether acupuncture at PC6 could be effective in preventing DOX-induced cardiotoxicity is still unknown.

**Methods:**

A set of experiments were performed with myocardial cells, wild type, inducible nitric oxide synthase knockout (iNOS-/-), and myocardial-specific ablation arginase 2 (Myh6-ARG 2-/-) mice. We investigated the protective effect and the underlying mechanisms for electroacupuncture (EA) against DOX-induced cardiotoxicity by echocardiography, immunostaining, biochemical analysis, and molecular biotechnology in vivo and in vitro analysis.

**Results:**

We found that DOX-mediated nitric oxide (NO) production was positively correlated with the iNOS level but has a negative correlation with the arginase 2 (ARG 2) level in both myocardial cells and tissues. Meanwhile, EA at PC6 alleviated cardiac dysfunction and cardiac hypertrophy in DOX-treated mice. EA at PC6 blocked the upregulation of NO production in accompanied with the downregulated iNOS and upregulated ARG 2 levels in myocardial tissue induced by DOX. Furthermore, knockout iNOS prevented cardiotoxicity and EA treatment did not cause the further improvement of cardiac function in iNOS-/- mice treated by DOX. In contrast, deficiency of myocardial ARG 2 aggravated DOX-induced cardiotoxicity and reduced EA protective effect.

**Conclusion:**

These results suggest that EA treatment at PC6 can prevent DOX-induced cardiotoxicity through modulating NO production by modulating the iNOS/ARG 2 balance in myocardial cells.

## 1. Introduction

Doxorubicin (DOX) is an anthracycline antibiotic that is widely used to treat leukemias, Hodgkin's lymphoma, cancers of the bladder and breast, multiple myeloma, and other cancers [1, 2]. However, DOX, like other anthracyclines, can damage the heart irreversibly. Cardiotoxicity characterized by decreased left ventricular ejection fraction, cardiomyopathy, and heart failure is a major side effect that can develop years after successful cancer therapy. At present, dexrazoxane is the only FDA-approved drug available to protect the heart against the cardiotoxic side effects of anthracyclines [3]. However, dexrazoxane is not used with the initiation of anthracycline therapy and is not approved for use in children or adolescents [4]. Thus, there is an urgent need for the development of effective and safe alternative therapies that can be used especially in earlier time windows or young patients to prevent anthracycline-induced cardiotoxicity.

Acupuncture including electroacupuncture (EA) is one of the most widely used and accepted complementary and alternative medical treatments. For cancer patients, many clinical studies have shown that acupuncture is helpful in alleviating side effects caused by chemotherapeutics, including pain, vomiting, fatigue, anxiety, insomnia, and postoperative intestinal obstruction [5–8]. The *Neiguan* acupoint (PC6) is located at the flexor aspect of the forearm between the tendons of the palmaris longus and flexor carpi radialis, overlying the median nerve [9]. PC6 stimulation has been used to improve symptoms of angina, palpitation, and left cardiac function in patients with heart diseases [10, 11]. Furthermore, experimental studies have reported that PC6 stimulation can effectively limit heart damage in various animal models including ischemia/reperfusion injury, myocardial ischemia, hypertension, hypertrophy, and bupivacaine-induced cardiotoxicity [12–15]. However, whether acupuncture at PC6 could be effective in preventing DOX-induced cardiotoxicity has not been reported yet.

The molecular mechanisms of DOX-induced cardiotoxicity include oxidative stress, calcium overload, lipid peroxidation, and mitochondrial dysfunction [16, 17]. Previous studies have suggested that increased nitric oxide (NO) production is involved in DOX-induced increases in nitrosative stress [18]. The production of NO is catalyzed by nitric oxide synthases (NOS) from L-arginine, and arginase competes with NOS for common substrates, L-arginine, to produce L-ornithine and urea [19]. In pathological conditions, the abnormal activity of inducible nitric oxide synthase (iNOS) or arginase disturbs the balance and causes the abnormal level of NO production [20]. Studies in models of DOX-induced cardiotoxicity have shown increased tissue levels of iNOS [21, 22]. Both two arginase isoforms, including arginases 1 and 2, are involved in the NO production in various cardiovascular diseases such as atherosclerosis and myocardial ischemia-reperfusion injury [23, 24]. Arginase 2 (ARG 2) is the predominant isoform expressed in cardiac tissue [25, 26]. However, the role of ARG 2 has not been reported in cardiomyocytes and further study is needed.

Some studies reported that EA can effectively alleviate hypertension and cardiac hypertrophy through regulating the NO level [27, 28]. Moreover, stimulation of PC6 can upregulate myocardial NO and NOS for relieving myocardial injury in myocardial ischemic reperfusion injury rats [29]. Therefore, we hypothesized that EA can provide cardioprotection against DOX-induced cardiotoxicity by regulating NO signaling. Here, we demonstrated DOX-induced cardiac injury was prevented by EA at PC6 through modulating the balance between iNOS and ARG 2 levels. These results strongly suggested that EA at PC6 should be seriously applied as an effective alternative therapy for protection against DOX-induced cardiotoxicity.

## 2. Materials and Methods

### 2.1. Experimental Animals

Eight-week-old male C57BL/6J mice were obtained from Jinan Pengyue Experimental Animal Breeding Co., Ltd. (Jinan, China). Knockout iNOS mice (iNOS-/-) were purchased from Jackson Laboratory (B6.129P2-Nos2tm1Lau/J, stock number: 002609). Myh6-CreER mice were purchased from Jackson Laboratory (stock number: 005657, B6129-Tg (Myh6-cre/Esr1)1Jmk/J). Floxed ARG 2 (ARG 2^f/f^) mice were generated by Shanghai Model Organisms Center, Inc. The mice were maintained in a 12 h light/dark cycle at 22 ± 2°C with relative humidity of 60% ± 5%. Food and water were supplied ad libitum. All experiments and animal care in this study were approved by the Institutional Animal Care and Use Committee of the Guangzhou University of Chinese Medicine.

### 2.2. DOX Administration

The DOX-induced cardiotoxicity animal model was established as previously described [30]. Briefly, mice were injected intraperitoneally with 3 mg/kg of DOX (D1515, Sigma, St. Louis, MO) for five continuous days; the vehicle control group received an equal volume of 0.9% NaCl.

### 2.3. Tamoxifen Administration

Tamoxifen administration was performed as we previously described [31]. Myh6-CreER mice were crossed with ARG 2^f/f^ mice to get Myh6-CreER; ARG 2^loxp/loxp^ (Myh6-ARG 2-/-). Tamoxifen (Sigma-Aldrich) was prepared in sesame oil (Sigma-Aldrich) and was intragastrically administered to the adult mice (100 mg/kg body weight/day, 8 weeks old) for 5 consecutive days.

### 2.4. EA Treatment

The intensity of EA was 2 mA, and the duration was 20 min per day for 7 continuous days, as described previously [32]. For EA treatment, mice were anesthetized with isoflurane (2%) and needles (0.18 mm × 7 mm, Suzhou Acupuncture & Moxibustion Appliance Co. Ltd., China) were inserted 2-3 mm into the left and right acupoints. Three groups of mice received EA. The frequency of the electric current of the EA therapeutic apparatus was set at 2, 50, or 100 Hz. To control for the influence of anesthesia in the EA groups, mice in the control and DOX groups were also given isoflurane (2%) inhalation anesthesia.

### 2.5. Experimental Design

To explore the effect of EA at PC6, mice were randomly divided into five groups as follows: the vehicle control group (Veh), DOX treatment group (DOX), and DOX with EA treatment group (DOX+2 Hz EA; DOX+50 Hz EA; DOX+100 Hz EA). Following treatments, mice were subjected to behavioral assessments and echocardiographic measurement. Then, mice were sacrificed to collect blood and heart tissue for biochemical tests. In some experiments, L-arginine (30 mg/mL, Sigma, USA) was administered in drinking water (130 mg/kg of body weight/day) as previously described [33]. For experiments using iNOS-/- mice, mice were randomly divided into five groups: control littermates with Veh and DOX treatments and iNOS-/- mice with Veh, DOX, and DOX+EA treatments. Experiments using ARG 2^f/f^ mice and Myh6-ARG 2^f/f^ mice were, respectively, divided into four groups: ARG 2^f/f^ with DOX treatment, ARG 2^f/f^ with DOX+EA treatment, Myh6-ARG 2-/- mice with DOX treatment, and Myh6-ARG 2-/- mice with DOX+EA treatment. The number and division of animals are shown in Supplementary Table 1-4.

### 2.6. Echocardiography

Echocardiography was performed as previously described [34]. Briefly, transthoracic echocardiography was performed using a high-resolution echo machine with a 30 MHz probe. Animals were anesthetized with 3% isoflurane, their chests were shaved, and temperature-controlled anesthesia was maintained with 1.5% isoflurane. The probe was situated perpendicular to the heart to determine the position, and then, the probe was rotated clockwise 30-45 degrees to determine the left ventricle of the heart. The following parameters were measured from the M-mode images and two-dimensional images obtained in the short-axis views by the corresponding matching software (Vevo 2100 high-resolution small animal ultrasound system, VisualSonics, Canada): the percentage of fractional shortening (FS, %), ejection fraction (EF, %), left ventricular end-diastolic volume (LVEDV), and left ventricular end-systolic volume (LVESV). Stroke volume (SV) was calculated using the formula: LVEDV − LVESV (Figure S1).

### 2.7. Rotarod Test

The rotarod test was performed as previously described [35]. Briefly, 12 days after DOX treatment, motor function was tested with a rotarod test (Shanghai Jiliang Software Technology Co. Ltd., China). The rotarod test was performed by placing a mouse on a rotating rod and measuring the time and distance it was able to maintain its balance. Mice were trained for 5 min three times in a day before the formal trial. The speed of the rotarod accelerated from 4 to 40 rpm over the 5 min period. To allow the mice to adapt to the accelerating rod, we put them back on the rod once they dropped from it during the training periods. In the formal trial, each mouse was placed on the rotating rod once and returned to the home cage after dropping from the rod. After an interval of 30 min, each mouse was subjected to another trial, for three trials in total.

### 2.8. Histological Analysis

Histological analysis was performed as previously described [36]. Hearts were excised after echocardiography, fixed overnight with 4% formalin/PBS-buffered, and embedded in paraffin. Transverse sections at a thickness of 5 *μ*m were cut and mounted on glass slides for hematoxylin and eosin (H&E) staining to evaluate gross morphology. Glass slides were dewaxed with xylene, followed by absolute alcohol and 95%, 85%, and 75% alcohol. The H&E staining sequence was as follows: hematoxylin for 6-8 min, washed by running water for 1 min; 0.2% hydrochloric alcohol for 3 s, washed by running water for another 1 min and soaked in double-distilled water for 10 min; eosin for 20-30 s, washed by xylene, absolute alcohol, and 75%, 85%, and 95% alcohol. To quantitate individual myocyte size, heart tissue sections were stained with FITC-conjugated wheat germ agglutinin (WGA) (Invitrogen, Carlsbad, CA, U.S.A.). The WGA staining sequence was as follows: incubation with WGA (10 *μ*g/mL) at room temperature for 20 min in darkness, washed in PBS 3 times for 5 min each; stained with 1 *μ*g/mL DAPI (4′,6-diamidino-2-phenylindole) for 10 min in darkness, washed in PBS 3 times for 5 min. For image analysis, the data value for each mouse was calculated from 5 sections/mouse and the number of mice in each group is 3.

### 2.9. Assessment of NO Production, Malondialdehyde (MDA), and 3-Nitrotyrosine (3-NT) Levels

Assessment of NO production was performed as previously described [37]. Pieces of frozen heart (~20 mg) were homogenized in precooled normal saline. The homogenate was centrifuged at 2000 r/min for 15 min at 4°C, and the supernatant was collected. NO production in the cardiac tissue was quantitated by evaluating its oxidation products (nitrate and nitrite) using the nitrate reductase method with a Total Nitric Oxide Assay Kit (No: S0024, Beyotime Institute of Biotechnology, Beijing, China) following the kit instructions. Briefly, the standard curve was obtained with NaNO_2_ at concentrations of 0, 1, 2, 5, 10, 20, 60, and 100 *μ*M. Samples (50 *μ*L/well) were mixed thoroughly with prewarmed Griess Reagent I and II (50 *μ*L of each reagent/well) in a 96-well plate. Then, the absorbance of each sample was determined at the wavelength of 540 nm. Total NO content (*μ*mol/g protein) was determined using a standard curve. MDA levels were determined using a MDA assay kit (Nanjing Jiancheng Bioengineering Institute Co., Ltd.; cat. no. A003-1-2) as previously described [38]. Simply, heart tissues were uniformly weighed and added corresponding reagents of the kit. Then, tubes were heated at 95°C for 40 min and centrifugated (4000 rpm, 10 min at 4°C). The OD values were measured at 532 nm. The concentrations of 3-NT were determined using a 3-NT assay kit (Elabscience Biotechnology Co., Ltd.) as previously described [39]. The OD values were measured at 450 nm. There are ten mice per group in animal experiments. All samples were assayed in triplicate.

### 2.10. Real-Time Quantitative PCR

Quantitative real-time PCR was performed as we previously described [40]. Briefly, total RNA was extracted from the heart tissue using Trizol reagent (9109, Takara, Japan) and complementary cDNA was synthesized using a PrimeScript™ RT reagent kit (RR047A, Takara, Japan). Gene expression of iNOS (forward, 5′-CGA GGA GGC TGC CTG CAG ACT TGG-3′ and 3′ reverse, 5′-CTG GGA GGA GCT GAT GGA GTA GTA-3′), endothelial nitric oxide synthase (eNOS) (forward, 5′-TCA GCC ATC ACA GTG TTC CC-3′ and reverse, 5′-ATA GCC CGC ATA GCG TAT CAG-3′), neuronal nitric oxide synthase (nNOS) (forward, ACCCAACGTCATTTCTGTCC and reverse, AAGGTGGTCTCCAGGTGTGT), TNF-*α* (forward, 5′-ACTCAACAAACTGCCCTTCTGAG-3′ and reverse, 5′-TTACAGCTG GTTTCGATCCATTT-3′), IL-1*β* (forward, 5′-TGTGGCTGTGGAGAAGCTGT-3′ and reverse, 5′-CAGCTCATATGGGTCCGAGA-3′), and IL-10 (5′-GTTGCCAAGCCTTATCGG GAA-3′ and 5′-CCAGGGAATTCAAATGCTCCT-3′) was determined by quantitative PCR with SYBR Green Dye Gene Expression Assays, performed on an ABI7500 system (Applied Biosystems, Carlsbad, CA, USA). The reaction conditions were as follows: 30 s polymerase activation at 95°C and 40 cycles at 95°C for 5 s, and 60°C for 31 s. *β*-Actin was used as internal control for normalization (forward, 5′-CTGACACCTTCACCATTCCAG-3′ and reverse, 5′-ATTGCTGACAGGATGCAG AAG-3′). The probes of TaqMan assay (Invitrogen) were used to detect ARG 2 and hypoxanthine phosphonbosyltransferase (HPRT) as internal control (Mm00477592_m1 and Mm00446968_m1). Data were normalized to HPRT, and the fold change between levels of different transcripts was calculated by the CT method. The number of mice in each group is 6, and each sample was tested in triplicate.

### 2.11. Arginase Activity

Heart tissues were used for arginase activity assay as described [40]. Briefly, 10 mM MnCl_2_ were added to the samples and heated at 57°C for 10 min to activate arginase. L-arginine (0.5 mol/L) was added and incubated at 37°C for 1 h. And the hydrolysis reaction was stopped with acid solution mixture (H_2_SO_4_: H_3_PO_4_: H_2_O). The solution of a-isonitrosopropiophenone (9%, a-ISPF in EtOH, Sigma, No: 13502) was added, and the mixture was heated at 100°C for 45 min. All samples were kept in the dark at room temperature for 10 min, and absorbance was measured by absorbance at 540 nm. There are ten mice per group in animal experiments and 6 samples per group in cell studies. All samples were assayed in triplicate.

### 2.12. Western Blot

Western blot was performed as we previously described [40]. Briefly, frozen cardiac samples were washed twice with cold PBS and resuspended in RIPA buffer. Equal amounts of total protein (30 *μ*g) were separated by SDS-PAGE. Then, gels were transferred to PVDF membranes and blocked for 1 h in blocking solution at room temperature. The membranes were incubated overnight at 4°C with primary antibodies (iNOS: 1 : 1000, ARG 2: 1 : 1000, Cell Signaling Technology, Danvers, MA, USA) followed by treatment with anti-rabbit secondary antibodies (1 : 5000, Cell Signaling Technology, Danvers, MA, USA) for 1 hour at room temperature. *β*-Tubulin (1 : 5000, Arigo Biolaboratories, Hsinchu City, Taiwan) was used as an internal control. An enhanced chemiluminescence ECL Plus system (Tanon, Shanghai, China) was used for visualization. There are six mice per group in animal experiments. All samples were assayed in triplicate.

### 2.13. Cell Culture and Treatment

Cell culture and treatment were performed as described [41]. Briefly, rat primary cardiomyocytes isolated from neonatal rat hearts (RAT-iCell-c001; iCell Bioscience, Shanghai, China) were cultured in a primary cardiomyocyte culture medium (PriMed-iCell-022; iCell Bioscience) in the incubator of 5% CO_2_ at 37°C. Rat primary cardiomyocytes were seeded at a density of 6.6 × 10^4^ cells/cm^2^ to incubate with DOX (0, 1.25, 2.5, 5, and 10 *μ*mol/L) for 24 h in darkness. Supernatant collection and protein extraction were performed after incubation for subsequent experiments. The measurement methods of NO production, arginase activity, ARG 2, and iNOS protein expression are the same as these of heart tissues.

### 2.14. Cell Viability

Cell viability was performed as described [42]. Rat primary cardiomyocytes were cultured in 96-well plates, and 10 *μ*L CCK-8 solution (Dojindo, Kumamoto, Japan) was added to each well at a 1/10 dilution, followed by a further 4 h incubation in the incubator. Absorbance was measured at 450 nm with a microplate reader (EPOCH, BioTek Winooski, Vermont, USA). Three wells in the indicated groups were used to calculate the percentage of cell viability according to the following formula: percentage of cell viability = (OD treatment group − OD blank control group)/(OD control group − OD blank control) × 100%. There are six samples per group, and experiments were repeated five times.

### 2.15. Statistical Analyses

All data were expressed as mean ± SEM. Statistical differences were determined using analysis of variance (ANOVA) followed by Turkey's post hoc test. The nonparametric Spearman rank correlation was calculated for NO production between iNOS and ARG 2 protein in cells. Comparisons were performed using SPSS (version 21.0). The number of experiments is indicated by “*n*,” and *P* values < 0.05 were taken as significant.

## 3. Results

### 3.1. DOX-Induced Cardiac Dysfunction Was Ameliorated by EA

**Figure 1:**
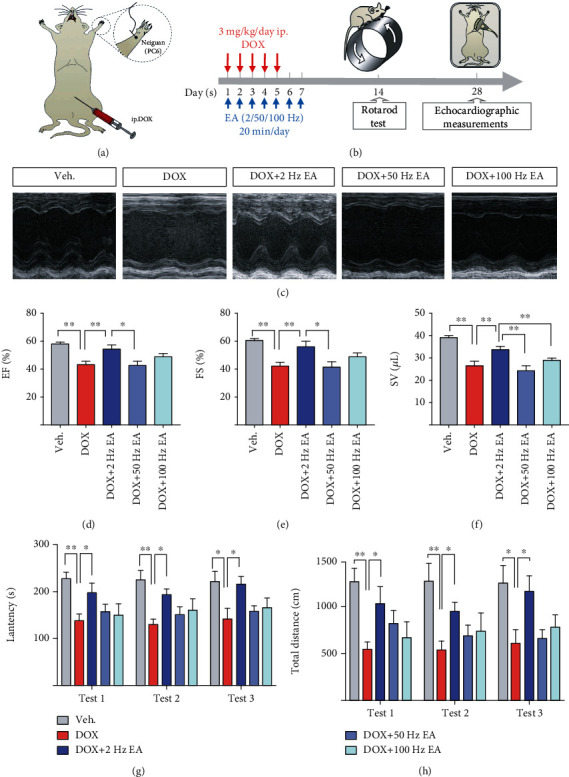
EA ameliorated DOX-induced heart dysfunction and impaired motor activity. (a) Illustration of the location of acupoint (*Neiguan*, PC6) and establishment of DOX-induced cardiotoxicity mice. (b) The experimental schedule of the DOX-induced cardiotoxicity model, EA treatment, rotarod test, and echocardiography. (c) Representative M-mode echocardiographic images. (d–f) Echocardiographic measurement of EF%, FS%, and SV. (g) Latency to fall from the rod (sec) and (h) total distance on the rod (cm) of the rotarod test in Veh and DOX-induced mice with and without EA treatment. Veh: mice treated with 0.9% NaCl; DOX: mice treated with DOX; DOX+2 Hz EA: DOX-induced mice treated with 2 Hz EA treatment; DOX+50 Hz EA: DOX-induced mice treated with 50 Hz EA treatment; DOX+100 Hz EA: DOX-induced mice treated with 100 Hz EA treatment; PC6: *Neiguan* acupoint; EA: electroacupuncture; DOX: doxorubicin; FS: fractional shortening; EF: ejection fraction; SV: stroke volume. Values are presented as mean ± SEM, ^∗^*P* < 0.05 and ^∗∗^*P* < 0.01, *n* = 8‐10 mice/group.

To investigate whether EA protected against DOX-induced cardiac dysfunction, we conducted echocardiography to measure the cardiac function in DOX-treated mice (Figure S1). Three different frequencies of EA stimulation at PC6 were performed for seven consecutive days (Figures 1(a) and 1(b)). As shown in Figures 1(c)–1(f), the values of EF%, FS%, and SV of the DOX group were significantly decreased, compared with the Veh group. Treatment of 2 Hz EA exhibited the best protection based on the values of EF%, FS%, and SV, compared with 50 Hz EA and 100 Hz EA treated groups (Figures 1(c)–1(f)). Previous studies have shown that impaired exercise ability is a manifestation of heart dysfunction [43]. To measure the motor function of mice, a rotarod test was used. As shown in Figures 1(g) and 1(h), the fall-off time and total distance on the rod decreased in the DOX group compared with the Veh group. EA at 2 Hz improved their motor function, but not 50 Hz EA or 100 Hz EA treatment. Taken together, these results suggested that DOX-induced cardiac dysfunction and impaired motor function were ameliorated by EA at 2 Hz.

### 3.2. DOX-Induced Cardiac Hypertrophy Was Alleviated by EA

**Figure 2:**
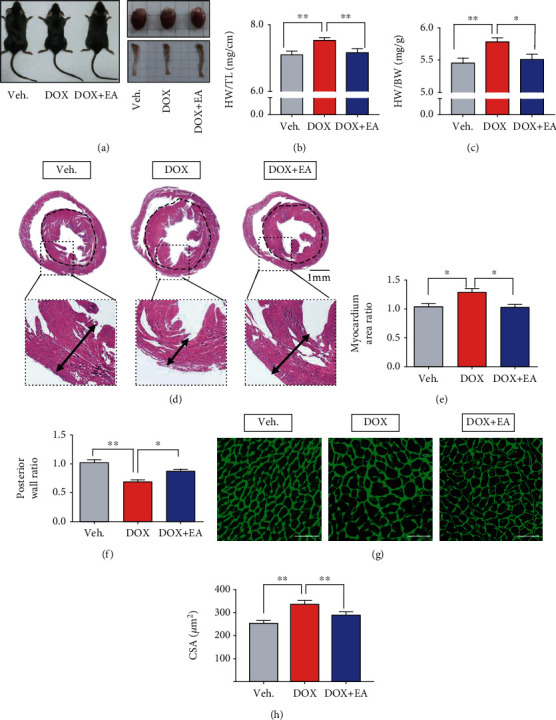
EA attenuated DOX-induced cardiac hypertrophy. (a) Photos of representative mice, heart and tibia samples of Veh, DOX, and DOX+EA groups. (b) The ratios of heart weight to tibia length (HW/TL) in each group. (c) The ratios of heart to body weight (HW/BW) in each group. (d) Representative images of heat sections by hematoxylin and eosin (H&E) staining in each group (scale bar = 1 mm). (e, f) Quantification of myocardium area ratio and posterior wall ratio of each heart section. (g) Representative images of heart sections by wheat germ agglutinin (WGA) staining in each group (scale bar = 50 *μ*m). (h) Quantification of cardiomyocyte size. Veh: mice treated with 0.9% NaCl; DOX: mice treated with DOX; DOX+EA: DOX-induced mice treated with 2 Hz EA treatment. Values are presented as mean ± SEM, ^∗^*P* < 0.05; ^∗∗^*P* < 0.01. *n* = 10 mice/group in a ratio of HW/TL and HW/BW. *n* = 3 mice/group in H&E and WGA staining.

Cardiac hypertrophy is one of the manifestations of DOX-induced cardiotoxicity [44]. To investigate the impact of DOX and EA treatment on pathological changes of the heart, we first measured heart weight and body weight. As shown in Figures 2(a)–2(c), the ratio of heart to body weight and heart weight to tibia length in the DOX group was increased compared with other groups. This result suggested that EA treatment prevented the DOX-induced cardiac hypertrophy. To further evaluate the effects of EA on DOX-induced cardiac hypertrophy, heart specimens were harvested at 20 days after DOX treatment for H&E and WGA staining. As shown in Figures 2(d)–2(f), the left ventricle chamber was larger and the ventricular posterior wall was thinner in the DOX group as compared with the control group. The EA treatment prevented these DOX-induced alterations. Consistently, DOX increased the size of the cardiomyocytes and this was also blocked by EA treatment (Figures 2(g) and 2(h)). These data indicated that DOX-induced hypertrophy was alleviated by EA at PC6.

### 3.3. EA Prevented DOX-Induced Cardiac Dysfunction through Regulation of NO Production

**Figure 3:**
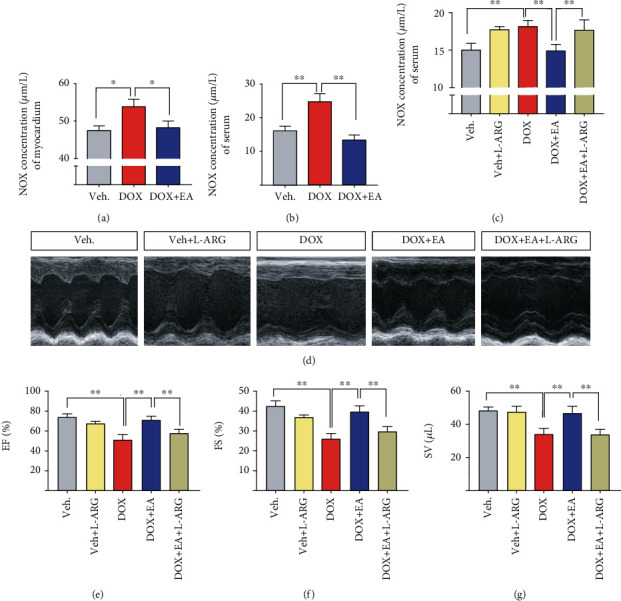
EA prevented DOX-induced cardiotoxicity through regulating the NO level. (a, b) NO concentration in heart tissue and serum of Veh, DOX, and DOX+EA groups. (c) NO concentration in serum of Veh, Veh+L-arginine (L-ARG), DOX, DOX+EA, and DOX+EA+L-ARG groups. (d) Representative M-mode echocardiographic images of the heart. (e–g) Echocardiographic measurement of cardiac EF%, FS%, and SV values in each group. Values are presented as mean ± SEM, ^∗^*P* < 0.05 and ^∗∗^*P* < 0.01. *n* = 6‐10 mice/group.

It has been reported that DOX-induced cardiotoxicity can be alleviated by modulating NO levels in the myocardium [45]. To determine whether EA treatment prevents DOX-induced heart dysfunction through an effect on NO, we first measured NO levels in serum and myocardium. We found that NO levels in both serum and heart were markedly increased in the DOX-treated group compared to the control group and this increase was blocked in the EA treatment group, suggesting that EA at PC6 prevented the DOX-induced increases in NO production (Figures 3(a) and 3(b)). Since L-arginine is the substrate used by NOS in the process of NO production [19], we treated mice with L-arginine supplement in DOX+EA groups. The purpose of this experiment is to determine whether the protective effect of EA was achieved by regulating the NO levels. First, we examined the impact of L-arginine on the increased NO level and cardiotoxicity induced by DOX. NO serum levels were analyzed in the five groups, which showed that the two groups with the administration of L-arginine had increased the NO level, compared with control and DOX+EA groups (Figure 3(c)). Next, we found that the values of FS%, EF%, and SV were decreased in the DOX+EA+L-ARG group, compared to the DOX+EA group, which suggested that the protective effect of EA was blocked by the increased NO production following the L-arginine administration (Figures 3(d)–3(g)). It was reported that NO was involved in the generation of oxidative/nitrosative stress [21]. Thus, we measured the levels of MDA and 3-NT in the heart tissues. Both levels of MDA and 3-NT were increased in DOX-induced mice significantly, which were prevented by EA treatment at PC6 (Figure S2). Increased levels of cardiac NO in DOX-induced heart may be activated by iNOS via inflammatory cytokines [46]. Next, we found increased cardiac inflammation including the changes of mRNA levels of TNF-*α*, IL-1*β*, and IL-10 in DOX-induced heart tissues, which were ameliorated by EA treatment as well (Figure S3). Together, these results indicated that a reduction in NO production is required for the therapeutic benefits of the EA treatment.

### 3.4. EA Modulated DOX-Induced Abnormal Levels of iNOS and ARG 2

**Figure 4:**
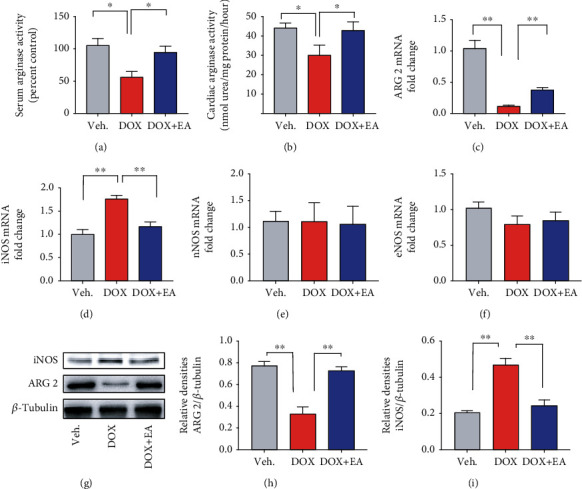
EA enhanced arginase activity and ARG 2 level in DOX-treated mice with reduced iNOS level. (a, b) The arginase activity in serum and cardiac tissues of Veh, DOX, and DOX+EA groups. (c–f) mRNA levels of ARG 2, iNOS, nNOS, and eNOS in cardiac tissues of Veh, DOX, and DOX+EA groups. (g) Representative images of ARG 2 and iNOS protein expression in cardiac tissues of each group. (h, i) Quantification of ARG 2 and iNOS protein expression in each group. *β*-Tubulin was used as the loading control. iNOS: inducible nitric oxide synthase; ARG 2: arginase 2; eNOS: endothelial nitric oxide synthase; nNOS: neuronal nitric oxide synthase. Values are presented as mean ± SEM, ^∗^*P* < 0.05 and ^∗∗^*P* < 0.01. *n* = 6 mice/group.

To further examine the role of NOS and arginase signaling in the effect of EA treatment, we next analyzed the arginase activity, expression levels of ARG 2, and three NOS isoforms including iNOS, eNOS, and nNOS in myocardial tissue. Both arginase activities in serum and myocardium were decreased significantly in the DOX group (Figures 4(a) and 4(b)), along with the reduced mRNA level of ARG 2 (Figure 4(c)). In contrast, the level of iNOS mRNA was increased in the DOX group compared to the control group (Figure 4(d)), but eNOS and nNOS were not altered (Figures 4(e) and 4(f)). Consistent with the changed mRNA level, there were lower ARG 2 levels and higher protein levels of iNOS in myocardial tissues of the DOX group (Figures 4(g)–4(i)). Furthermore, EA treatment at PC6 reversed the DOX-induced abnormal levels of iNOS and ARG 2 (Figures 4(a)–4(d) and 4(g)–4(i)). These results indicated that DOX administration caused both abnormal levels of iNOS and ARG 2, which can be restored by EA at PC6.

### 3.5. DOX Induced the Increased iNOS but Decreased ARG 2 Levels in Myocardial Cells

**Figure 5:**
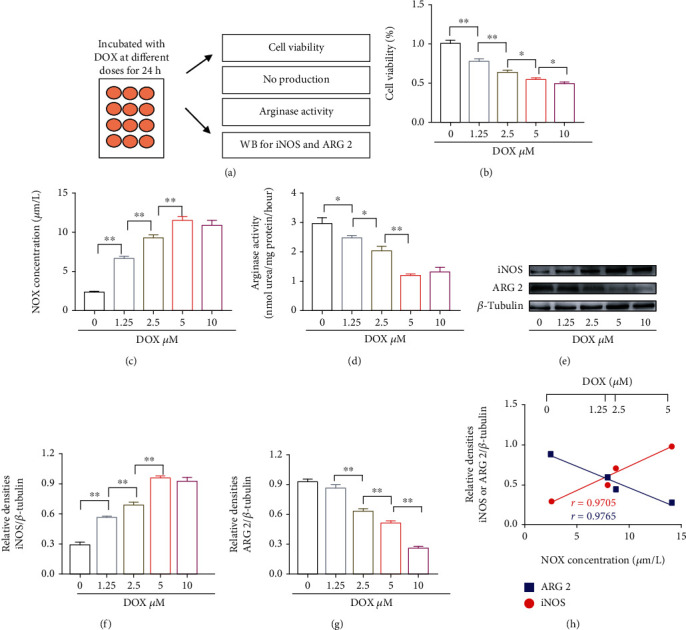
Both iNOS and ARG 2 are implicated in the DOX-induced cytotoxicity in myocardial cells. (a) The schematic diagram of rat embryonic ventricular myocardial cells with DOX inducement. (b) The value of cell viability, (c) NO production, and (d) arginase activity under the various concentrations of DOX inducement (1.25, 2.5, 5, and 10 *μ*M) in rat primary cardiomyocytes. (e) Presentative images of iNOS and ARG 2 protein expression in rat primary cardiomyocytes with and without DOX inducement (0, 1.25, 2.5, 5, and 10 *μ*M), and *β*-tubulin was used as the loading control. (f, g) Quantification of iNOS and ARG 2 protein expression in all groups. (h) Correlation analysis of iNOS and ARG 2 protein expression to NO production, respectively, in all groups. Values are presented as mean ± SEM, ^∗^*P* < 0.05 and ^∗∗^*P* < 0.01. *n* = 6 samples/group.

To further clarify whether cardiomyocyte ARG 2 and iNOS are involved in the DOX-induced cytotoxicity, we performed a set of experiments in rat primary cardiomyocytes (Figure 5(a)). Exposure of myocardial cells to the different concentrations of DOX (0, 1.25, 2.5, 5, and 10 *μ*M) for 24 h caused a significant reduction of cell viability and arginase activity with the enhancement of NO production in a dose-dependent way (Figures 5(b)–5(d)). Furthermore, all different concentrations of DOX significantly increased iNOS and reduced ARG 2 protein levels of myocardial cells compared with controls (Figures 5(e)–5(g)). As shown in Figure 5(h), the DOX-mediated level of NO production was positively correlated with iNOS levels (*r* = 0.9705), but negatively correlated with ARG 2 protein levels (*r* = 0.9765) at different concentrations of DOX stimulation (0, 1.25, 2.5, and 5 *μ*M). Together, the above results suggested that both alterations of cardiomyocyte iNOS and ARG 2 levels can contribute to the abnormal NO production induced by DOX.

### 3.6. iNOS Was Critical for Protection of EA against DOX-Induced Cardiac Dysfunction

**Figure 6:**
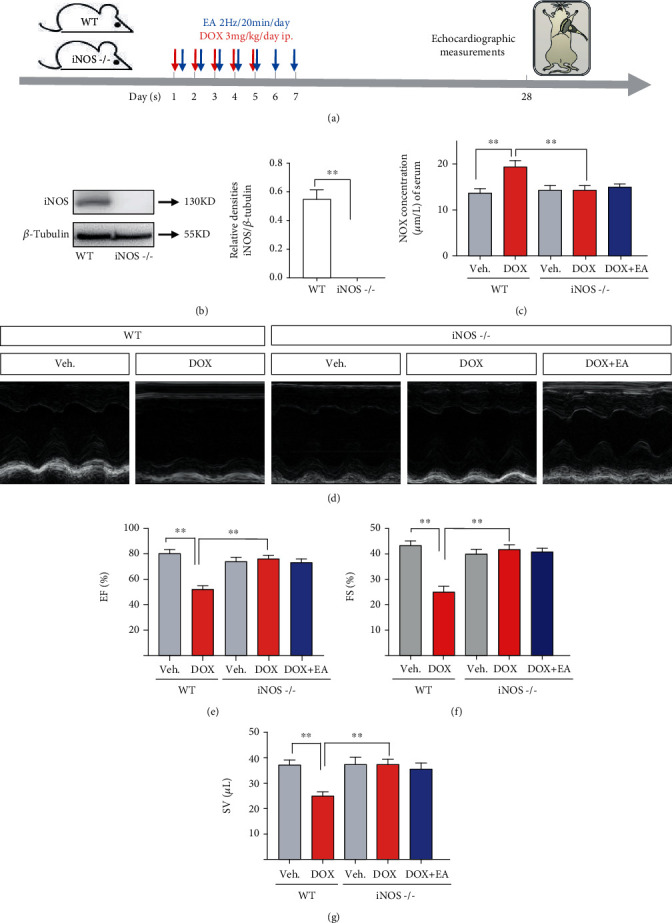
Prevention of EA in DOX-induced cardiotoxicity was blocked by knocking out iNOS. (a) The experimental schedule of DOX-induced cardiotoxicity model, EA treatment, and echocardiography measurement in wild-type (WT) mice and iNOS knocking out (iNOS-/-) mice. (b) The protein expression of iNOS in WT and iNOS-/- hearts; (c) NO concentration of serum in Veh and DOX-induced WT and iNOS-/- mice with and without EA treatment; (d) representative M-mode echocardiographic images of hearts; (e–g) echocardiographic measurement of cardiac EF%, FS%, and SV values in each group. Values are presented as mean ± SEM, ^∗^*P* < 0.05 and ^∗∗^*P* < 0.01, *n* = 6 mice/group.

To further examine the role of iNOS in DOX-induced heart dysfunction and EA treatment, we performed experiments using iNOS-/- mice (Figure 6(a)). First, we confirmed that the iNOS protein level was undetectable in iNOS-/- mice (Figure 6(b)). As shown in Figure 6(c), knocking out of iNOS blocked the NOX-induced increase in NO production. We also found that EA treatment did not alter the levels of NO in the iNOS-/- mice with and without DOX administration. Next, we performed echocardiographic measurement to evaluate heart function in the different groups. Unlike the DOX-treated wildtype control which showed significant decreases in EF%, FS%, and SV, iNOS-/- mice with the DOX treatment showed no changes in the above three test indexes. This data suggests that knocking out iNOS can prevent the DOX-induced cardiac dysfunction (Figures 6(d)–6(g)). Considering that both EA treatment and iNOS knockout prevent DOX-induced heart impairment, we then determined whether they act via the same mechanism. If so, EA should not be able to further improve the heart function in the DOX-treated iNOS-/- mice. Consistent with this hypothesis, EA at PC6 failed to further increase the values of EF%, FS%, and SV in iNOS-/- mice, compared with DOX-treated iNOS-/- mice (Figures 6(d)–6(g)). Together, these results indicated that iNOS is a therapeutic target for the prevention of EA treatment against DOX-induced cardiotoxicity.

### 3.7. Ablation of Cardiomyocyte-Specific ARG 2 Exacerbated DOX-Induced Cardiac Dysfunction and Weakened the Protective Effect of EA Treatment

**Figure 7:**
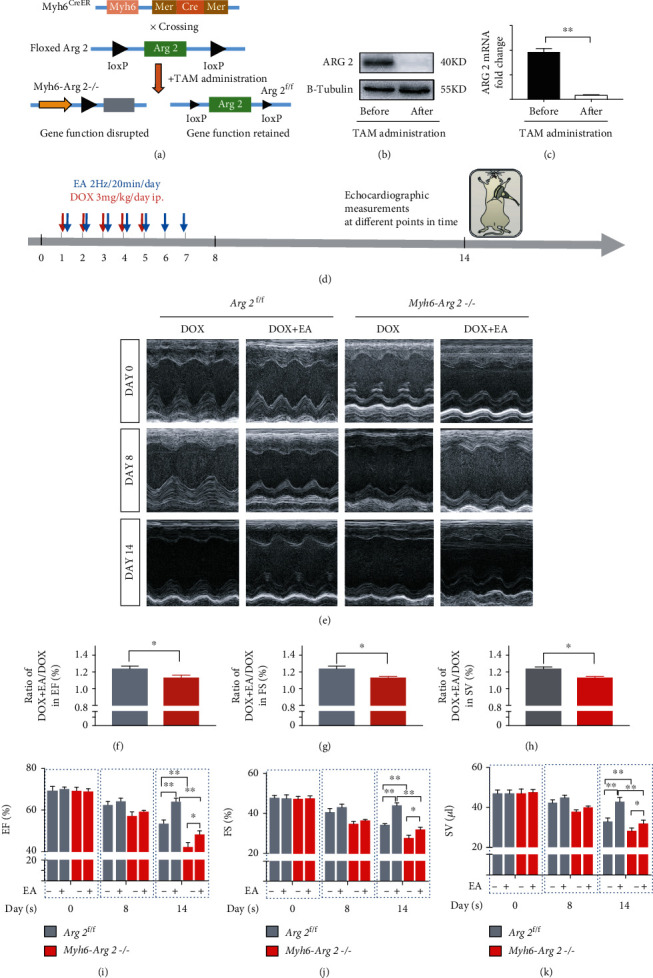
Deficiency of ARG 2 in the heart weakened the protective effect of EA in DOX-induced cardiac dysfunction. (a) Mating strategy of Myh6-ARG 2-/- mice from ARG 2^f/f^ and Myh6-CreER mice; (b, c) ARG 2 protein and mRNA levels in myocardial tissue of ARG 2^f/f^ and Myh6-ARG 2-/-mice; (d) the experimental schedule of DOX administration, EA treatment, and echocardiographic measurement in ARG 2^f/f^ and Myh6-ARG 2-/- mice; (e) representative M-mode echocardiographic images in DOX-induced ARG 2^f/f^ and Myh6-ARG 2-/- mice with and without EA treatment at 0, 8, and 14 days of DOX injection; (f–h) ratios of DOX+EA to DOX in the values of EF%, FS%, and SV in DOX-induced ARG 2^f/f^ and Myh6-ARG 2-/- hearts; (i–k) echocardiographic measurement of EF%, FS%, and SV values at three different points of time in DOX-induced ARG 2^f/f^ and Myh6-ARG 2-/- mice with and without EA treatment. Values are presented as mean ± SEM, ^∗^*P* < 0.05 and ^∗∗^*P* < 0.01, *n* = 8‐12 mice/group.

The change of ARG 2 and iNOS levels presented an opposite trend in both DOX-induced cardiomyocytes and cardiac tissues (Figures 4 and 5). Meanwhile, arginase negatively regulated NO production by competing with NOS for their common substrate L-arginine. Therefore, we hypothesized that DOX induced cardiotoxicity through breaking the ARG 2/iNOS balance. If so, ablation of cardiomyocyte-specific ARG 2 should exacerbate DOX-induced cardiac dysfunction and weaken the protective effect of EA treatment. To this end, ARG 2^f/f^ mice were crossed with Myh6-CreER mice to generated mice lacking ARG 2 in myocardial cells until TAM administration (Myh6-ARG 2-/-) (Figure 7(a)). As shown in Figures 7(b) and 7(c), ARG 2 protein and mRNA level in myocardial tissues were markedly reduced in Myh6-ARG 2-/- mice, compared to ARG 2^f/f^ mice. Both ARG 2^f/f^ and Myh6-ARG 2-/-mice have received DOX injection for 5 days and EA treatment for 7 days from the same day; echocardiographic measurements were performed at 0, 8, and 14 days of DOX injection (Figure 7(d)). As Figures 7(i)–7(k) show, Myh6-ARG 2-/- mice exhibited the same cardiac function before DOX treatment and the significant impairment of heart function at 14 days from the first DOX-injection, compared with ARG 2^f/f^ mice. Furthermore, it is worth noting that the deficiency of ARG 2 in myocardial cells reduced the degree of improvement of EA on DOX-induced cardiac dysfunction, which was presented by the lower ratios of DOX+EA to DOX in the values of EF%, FS%, and SV from Myh6-ARG 2-/- mice, compared with ARG 2^f/f^ mice (Figures 7(f)–7(h)). All results suggested that myocardial ARG 2 is also critical in DOX-induced heart dysfunction and in the protection of EA against DOX-induced cardiotoxicity.

## 4. Discussion

*Neiguan* (PC6) acupoints have been reported to effectively improve cardiac function in various diseases [10]. In this study, EA at PC6 significantly improved left ventricular systolic dysfunction and cardiac hypertrophy and increased motor ability in mice with DOX-induced cardiotoxicity (Figures 1 and 2). Consistent with our results, one study showed that puncturing acupoints PC6 and PC4 (*Ximen*) enhanced the contractility of the left ventricle wall and increased the stroke volume of the heart in patients with coronary heart disease [47]. In addition, a randomized clinical trial showed that EA pretreatment at PC6 and PC4 reduced myocardial injury after postpercutaneous coronary intervention in patients with coronary artery diseases [48]. Moreover, acupuncture at PC6 improved cardiac function in a myocardial ischemia rat model by increasing left ventricular diastolic and systolic function [14]. Similarly, acupuncture at PC6 prevented myocardial hypertrophy [49] and improved the value of FS% in mice with cardiac hypertrophy [13]. Our results provide evidence for the first time that stimulation at PC6 is an effective therapy to prevent DOX-induced cardiotoxicity.

The appropriate frequency of EA stimulation at PC6 for treatment of DOX-induced cardiotoxicity is still unclear. Our results provide evidence to show that 2 Hz is the most effective level of EA in DOX-treated mice compared with 50 Hz and 100 Hz (Figure 1). Similarly, it was reported that the application of low-frequency EA significantly improved heart rate variability, sympathetic stress, and parasympathetic vagal tone in patients [50]. Compared with the stimulation frequency of 40 or 100 Hz, percutaneous EA at the PC5 and PC6 acupoints with 2 Hz protected against the stress-induced myocardial ischemia [51]. In addition, low-frequency (1-5 Hz) EA inhibited the reflex cardiovascular pressor response via inhibiting sympathetic excitation [52]. The underlying mechanism for this was unknown, but one study showed that the somatic sensory nerve fiber stimulation underlies acupuncture's cardiovascular actions and low-frequency EA can lead to much greater activation of afferent fibers than higher stimulation frequencies [53]. In other diseases including chronic pain and erectile dysfunction in a rat model, low-frequency EA also proved more effective in treating different diseases [54, 55].

NO is a small signaling molecule that is critically involved in cell growth, differentiation, and apoptosis. Our results showed that DOX-induced cardiotoxicity in mice is associated with increased levels of NO, oxidative/nitrosative stress, and inflammation of myocardium (Figure 3, Figures S2 and S3), consisting with the conclusion in another study that DOX-induced cytotoxicity is due to an increase in oxidative/nitrosative stress and inflammation [18]. Furthermore, our study found that the EA at PC6 treatment improved heart function in DOX-treated mice by a mechanism involving reductions in NO levels (Figure 3). Consistent with our results, decreased NO serum level resisted DOX-induced cardiotoxicity [56]. Studies have shown that the DOX-induced damage of cardiac tissue can be prevented through reductions in activities of NOS [57]. The expression of the inducible NOS isoform iNOS is frequently associated with inflammation and malignant diseases [58]. Previous studies have validated the cardioprotective effect of iNOS inhibition in DOX-mediated cardiotoxicity [21, 22]. Consistently, we found that EA at PC6 decreased the expression of iNOS in heart tissue in DOX mice (Figure 4). Our data also showed that knocking out iNOS can protect against DOX-induced cardiotoxicity. However, the protective effect protection was not further improved by combination with EA treatment (Figure 6). This suggests that the protective effect of EA at PC6 on DOX-induced cardiotoxicity occurs via reducing the expression of iNOS.

**Figure 8:**
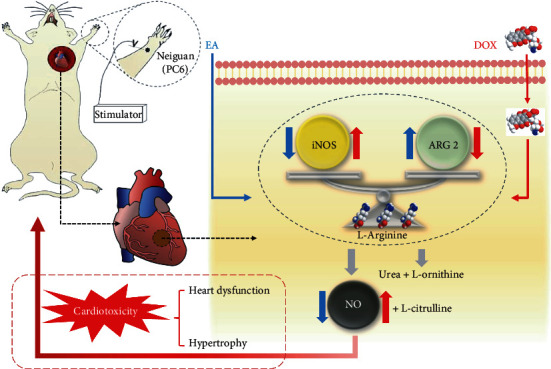
A proposed mechanism underlying DOX-induced cardiotoxicity and EA treatment. DOX inducement caused the abnormal NO signaling including increased ARG 2 and decreased iNOS levels in myocardial cells. EA prevented against DOX-induced heart dysfunction and hypertrophy by regulating cardiomyocyte iNOS/ARG 2 balance.

Previous studies supported that the treatment of diseases in TCM is through the downregulation of “hyperactive” signaling pathways and the upregulation of “deficient” signaling pathways, which can balance and restore the normal status [59]. Excessive arginase is highly involved in the regulation of NO production by competing with NOS for L-arginine, causing NOS uncoupling in several disease models [60]. Similarly, we found that the abnormal increasing NO in DOX-induced cardiomyocytes was caused by the imbalance between the expression of iNOS and ARG 2 in this study (Figure 8). Consistently, other studies reported that the competition of the common substrate between arginase and eNOS affected NO production and further caused endothelial dysfunction condition, which can be improved by adjusting both ARG 2 and eNOS signaling [61, 62]. Additionally, it was worthy to point out that EA treatment still ameliorated the damage of the heart due to the ablation of myocardial ARG 2, which suggested EA ameliorating DOX-induced cardiotoxicity through other signaling besides the ARG 2 pathway, which needs to be further investigated.

## 5. Conclusions

In the current study, we found that EA at PC6 with 2 Hz effectively protected against DOX-induced cardiotoxicity and overactivated iNOS-NO signaling. Second, cardiomyocyte-specific ARG 2 exacerbated DOX-induced cardiotoxicity accompanied with abnormal NO production and iNOS increased. Third, genetically intervention iNOS reduced the DOX-induced cardiotoxicity and EA treatment in iNOS-/- mice did not cause the further improvement on the impaired cardiac function. In contrast, ablation myocardial ARG 2 exacerbated DOX-induced cardiac dysfunction and weakened the protective effect of EA treatment. These results revealed that the cardioprotection of EA treatment against DOX-induced cardiotoxicity is through modulating the iNOS/ARG 2 balance and restoring the impaired NO production in cardiomyocytes.

## Data Availability

The data used to support the findings of this study are available from the corresponding author upon request.

## Supplementary Materials

Supplementary Materials Figure S1: schematic diagram of the measurement of mouse left ventricle function by ultrasound system and the calculation of parameters. An illustration on the left is B-mode ultrasound of the short axis section in the left ventricle, and an orange single scan line is placed next to the papillary muscles. An illustration on the right is M-mode ultrasound recording the left ventricular motion curve in the area marked by an orange single scan line. The measurement parameters included left ventricular end-diastolic diameter (LVID; d) and end-systolic diameter (LVID; s), left ventricular end-diastolic anterior wall thickness (LVAW; d), end-systolic anterior wall thickness (LVAW; s), left ventricular end-diastolic posterior wall thickness (LVPW; d), and left ventricular end-systolic posterior wall thickness (LVPW; s). The above parameters are calculated by Vevo 2100 to get left ventricular ejection fraction (EF%), shortening rate of left ventricular short axis (FS%), left ventricular end-diastolic volume (LVEDV), and left ventricular end-systolic volume (LVESV). Stroke volume (SV) was calculated as follows: SV = LVEDV − LVESV. Figure S2: EA treatment prevented abnormal levels of cardiac oxidative/nitrosative stress in DOX-induced mice. (A) The cardiac MDA level in each group; (B) the cardiac 3-NT level in each group; Veh: mice treated with 0.9% NaCl; DOX: mice treated with DOX; DOX+EA: DOX-induced mice treated with 2 Hz EA treatment. MDA: malondialdehyde; 3-NT: 3-nitrotyrosine.Values are presented as mean ± SEM, ^∗∗^*P* < 0.01. *n* = 10 mice/group. Figure S3: EA treatment reduced cardiac inflammation levels in DOX-induced mice. (A-C) mRNA levels of TNF-*α*, IL-1*β*, and IL-10 in heart tissues of Veh, DOX, and DOX+EA groups. Veh: mice treated with 0.9% NaCl; DOX: mice treated with DOX; DOX+EA: DOX-induced mice treated with 2 Hz EA treatment. Values are presented as mean ± SEM, ^∗^*P* < 0.05; ^∗∗^*P* < 0.01. *n* = 10 mice/group. Supplementary Table 1: experiment for investigating the best frequency of EA treatment. Supplementary Table 2: experiment for investigating the production of NO in the efficacy of EA. Supplementary Table 3: experiment for investigating the role of iNOS in the efficacy of EA. Supplementary Table 4: experiment for investigating the role of ARG 2 in the efficacy of EA.
