# Supplementary material for: Protection against Doxorubicin-Induced Cardiotoxicity through Modulating iNOS/ARG 2 Balance by Electroacupuncture at PC6
Source: Oxid Med Cell Longev. 2021 Mar 20;2021:6628957. doi: 10.1155/2021/6628957 (PMC8007344; doi:10.1155/2021/6628957)
Supplement: Supplementary Materials — Figure S1: schematic diagram of the measurement of mouse left ventricle function by ultrasound system and the calculation of parameters. An illustration on the left is B-mode ultrasound of the short axis section in the left ventricle, and an orange single scan line is placed next to the papillary muscles. An illustration on the right is M-mode ultrasound recording the left ventricular motion curve in the area marked by an orange single scan line. The measurement parameters included left ventricular end-diastolic diameter (LVID; d) and end-systolic diameter (LVID; s), left ventricular end-diastolic anterior wall thickness (LVAW; d), end-systolic anterior wall thickness (LVAW; s), left ventricular end-diastolic posterior wall thickness (LVPW; d), and left ventricular end-systolic posterior wall thickness (LVPW; s). The above parameters are calculated by Vevo 2100 to get left ventricular ejection fraction (EF%), shortening rate of left ventricular short axis (FS%), left ventricular end-diastolic volume (LVEDV), and left ventricular end-systolic volume (LVESV). Stroke volume (SV) was calculated as follows: SV = LVEDV − LVESV. Figure S2: EA treatment prevented abnormal levels of cardiac oxidative/nitrosative stress in DOX-induced mice. (A) The cardiac MDA level in each group; (B) the cardiac 3-NT level in each group; Veh: mice treated with 0.9% NaCl; DOX: mice treated with DOX; DOX+EA: DOX-induced mice treated with 2 Hz EA treatment. MDA: malondialdehyde; 3-NT: 3-nitrotyrosine.Values are presented as mean ± SEM, ∗∗P < 0.01. n = 10 mice/group. Figure S3: EA treatment reduced cardiac inflammation levels in DOX-induced mice. (A-C) mRNA levels of TNF-α, IL-1β, and IL-10 in heart tissues of Veh, DOX, and DOX+EA groups. Veh: mice treated with 0.9% NaCl; DOX: mice treated with DOX; DOX+EA: DOX-induced mice treated with 2 Hz EA treatment. Values are presented as mean ± SEM, ∗P < 0.05; ∗∗P < 0.01. n = 10 mice/group. Supplementary Table 1: experiment for investigating the best fre [file 6628957.f1.docx]

## Supplementary Materials


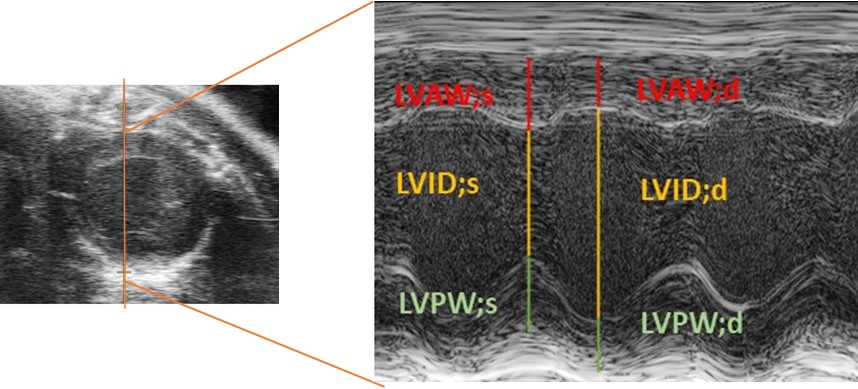


**Figure S1**. **Schematic diagram of the measurement of mouse left ventricle function by ultrasound system and the calculation of parameters.** An illustration on the left is B-mode ultrasound of the short axis section in the left ventricle and an orange single scan line is placed next to the papillary muscles. An illustration on the right is M-mode ultrasound recording the left ventricular motion curve in the area marked by orange single scan line. The measurement parameters included left ventricular end diastolic diameter (LVID;d) and end systolic diameter (LVID;s), left ventricular end diastolic anterior wall thickness (LVAW;d), end systolic anterior wall thickness (LVAW;s), left ventricular end diastolic posterior wall thickness(LVPW;d) and left ventricular end systolic posterior wall thickness(LVPW;s). The above parameters are calculated by Vevo2100 to get left ventricular ejection fraction (EF%), shortening rate of left ventricular short axis (FS%), left ventricular end diastolic volume (LVEDV) and left ventricular end systolic volume (LVESV). Stroke volume (SV)was calculated as follows: SV= LVEDV- LVESV.


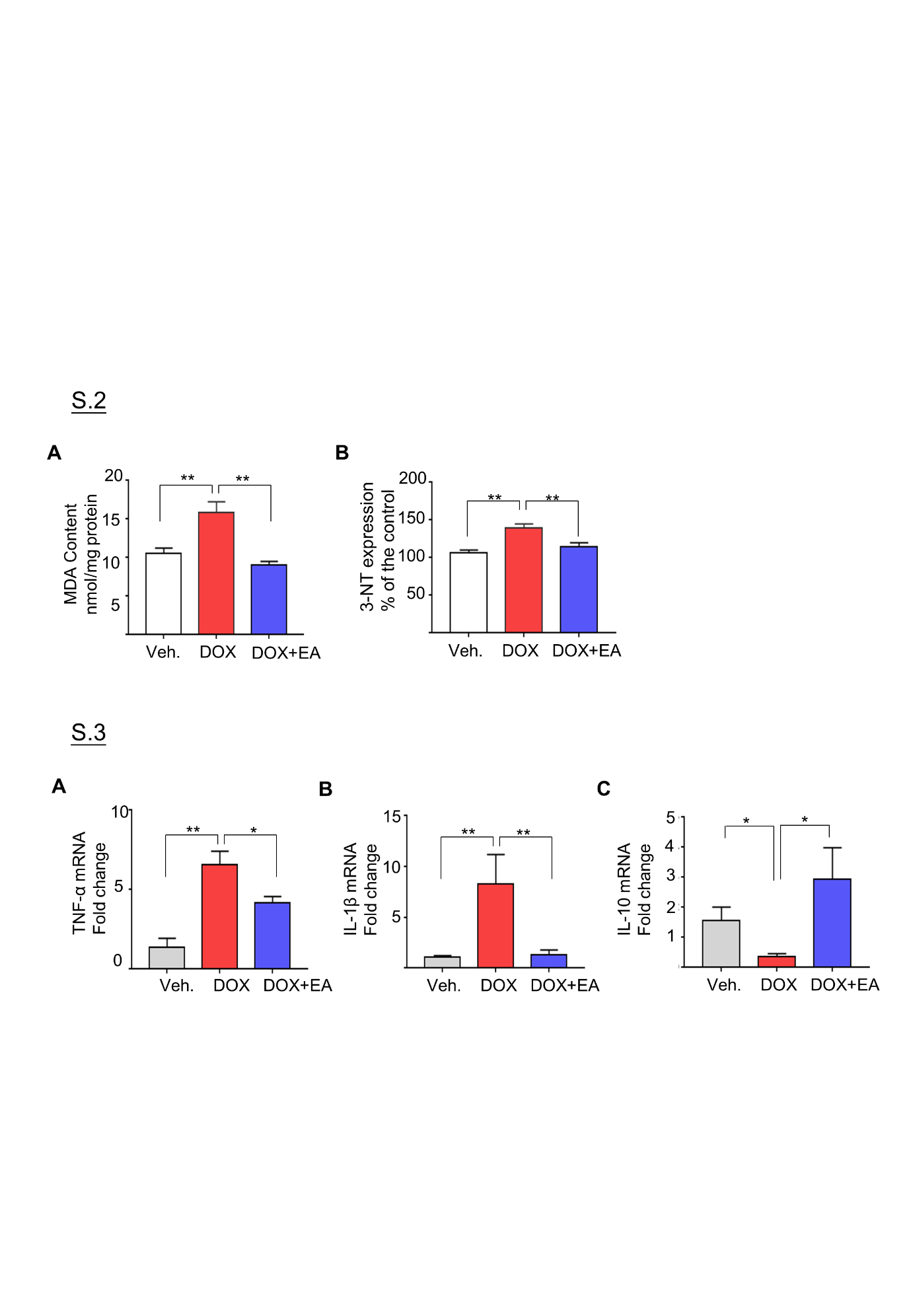


**Figure S2**.  **EA treatment prevented abnormal levels of cardiac oxidative/nitrosative stress in DOX-induced mice. (A)** The cardiac MDA level in each group; **(B)** The cardiac 3-NT level in each group; Veh: mice treated with 0.9% NaCl; DOX: mice treated with DOX; DOX+ EA: DOX-induced mice treated with 2 Hz EA treatment. MDA: malondialdehyde; 3-NT: 3-Nitrotyrosine.Values are presented as mean ± SEM, ***P*<0.01. n=10 mice / group.


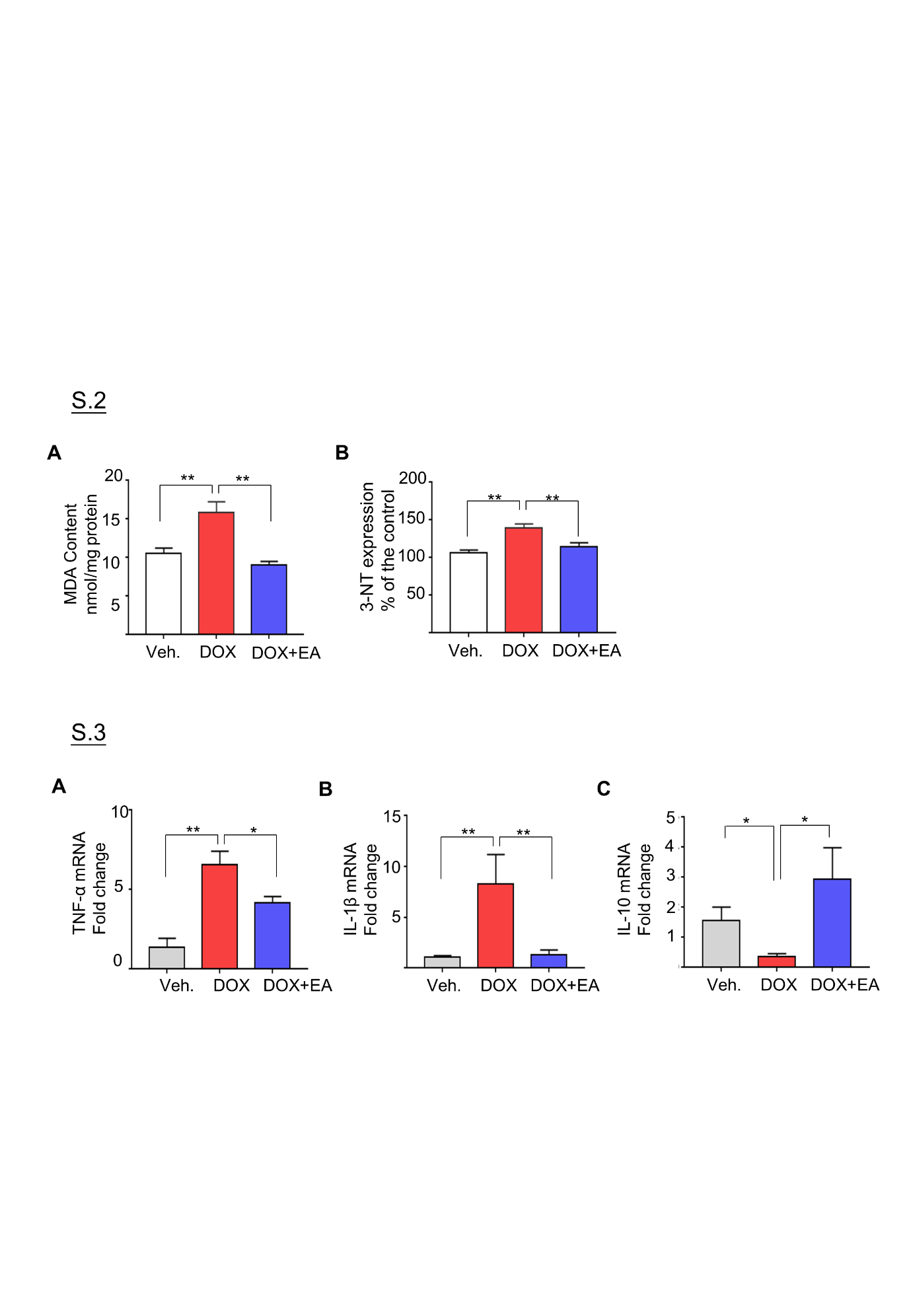


**Figure S3**. **EA treatment reduced cardiac inflammation levels in DOX-induced mice. (A-C)** mRNA levels of TNF-α, IL-1β and IL-10 in heart tissues of Veh, DOX and DOX+EA groups**.** Veh: mice treated with 0.9% NaCl; DOX: mice treated with DOX; DOX+ EA: DOX-induced mice treated with 2 Hz EA treatment. Values are presented as mean ± SEM,**P*<0.05; ***P*<0.01. n=10 mice / group.

Supplementary Table 1: Experiment for investigating the best frequency of EA treatment

| Group | Strain | Treatment | No. of animal |  |
| --- | --- | --- | --- | --- |
| Control | C57BL/6J | 0.9%NaCl(ip.) | 10 |  |
| DOX | C57BL/6J | DOX (3mg/kg/day, ip.) | 10 |  |
| DOX+2 Hz EA | C57BL/6J | DOX+2 Hz EA | 10 |  |
| DOX+50 Hz EA | C57BL/6J | DOX+50 Hz EA | 8 |  |
| DOX+100 Hz EA | C57BL/6J | DOX+100 Hz EA | 8 |  |

Supplementary Table 2: Experiment for investigating the production of NO in the efficacy of EA.

| Group | Strain | Treatment | No. of animal |  |
| --- | --- | --- | --- | --- |
| Control | C57BL/6J | 0.9%NaCl (ip.) | 9 |  |
| Control +L-ARG | C57BL/6J | 0.9%NaCl+L-ARG (130mg/kg, drinking water) | 6 |  |
| DOX | C57BL/6J | DOX (3mg/kg/day, ip.) | 10 |  |
| DOX+2 Hz EA | C57BL/6J | DOX+2 Hz EA | 10 |  |
| DOX+2 Hz EA+L-ARG | C57BL/6J | DOX+2 Hz EA+L-ARG | 10 |  |

Supplementary Table 3: Experiment for investigating the role of iNOS in the efficacy of EA.

| Strain | Gene type | Treatment | No. of animal |  |
| --- | --- | --- | --- | --- |
| iNOS | WT | 0.9%NaCl(ip.) | 6 |  |
| iNOS | WT | DOX (3mg/kg/day, ip.) | 6 |  |
| iNOS | KO | 0.9%NaCl | 6 |  |
| iNOS | KO | DOX | 6 |  |
| iNOS | KO | DOX+2 Hz EA | 6 |  |

Supplementary Table 4: Experiment for investigating the role of ARG2 in the efficacy of EA.

| Strain | Gene type | Treatment | No. of animal |  |
| --- | --- | --- | --- | --- |
| Myh6-Arg2 | Arg2 f/f | DOX (3mg/kg/day, ip.) | 12 |  |
| Myh6-Arg2 | Arg2 f/f | DOX+2 Hz EA | 10 |  |
| Myh6-Arg2 | Myh6-ARG 2-/- | DOX | 8 |  |
| Myh6-Arg2 | Myh6-ARG 2-/- | DOX+2 Hz EA | 10 |  |
